# Two Interesting Cases Reported at the Annual Meeting of the West Texas Medical Association, Held in San Antonio, Texas, October, 27th, 1898*Read at annual meeting of West Texas Medical Society at San Antonio.

**Published:** 1898-11

**Authors:** Alfred C. M’Daniel

**Affiliations:** San Antonio, Texas


For Texas Medical Journal.

### Two Interesting Cases Reported at the Annual Meeting of the West Texas Medical Association, Held in San Antonio, Texas, October, 27th, 1898.*

BY ALFRED C. M'DANIEL, M. D., SAN ANTONIO, TEXAS.

The first case which I wish to report this evening is one of osteomyelitis of the femur.

The patient is a boy seven years old. Family and personal history both good. No prodromal symptoms. Boy is very playful and always fond of running and jumping off high places; but gives no history of actual injury.

During the first part of May of this year, on an afternoon, he complained of pain in his left knee and laid down on the bed. When supper time came, the knee hurt him so badly that he asked his father to carry him out to the table. He did not walk from that time until quite recently. The patient was then at his home in an adjoining town and a local physician was called in to see him.

I am told that the knee and leg were intensely painful, red and a little swollen. The attending physician pronounced it a case of acute rheumatism and applied ice externally and gave various medicines internally. In a few days the leg rapidly became larger and the swelling and sorenes extended upward.

The fever varied from 101 F. to 103 F. He was thus treated under the diagnosis of rheumatism for five or six weeks, when he was brought to San Antonio, where he was seen by one of our surgeons and on the same day by another one, who made a small opening in the skin and, according to the family's statement, emptied one or more quarts of pus from the leg. The cavity was then washed out frequently, and a day or two later the attending surgeon made a counter opening on the opposite side of the leg and washed through and through.

It was at this stage that the case, after having been sick for about seven weeks, came into my hands. At that time, his morning temperature was 102 2-5 deg. F.—pulse very rapid and weak—appetite enormous—movements of the bowels dysenteric—patient nervous, anæmic and in a bad condition generally.

Dr. Edmondson saw the case with me and we both thought that

*Read at annual meeting of West Texas Medical Society at San Antonio.

an early operation offered the best chance for life and recovery; so he was at once sent to the hospital.

His dysentery became so bad, however, that I thought it advisable to diet him and postpone operating for a few days, which I did.

On July 6th, I, with the assistance of Drs. Edmondson, Earp and Luter, chloroformed the patient and enlarged the small incision which had previously been made in the skin. The pus which had formerly accumulated had dissected the muscles from the femur for a distance of about nine inches and a very large cavity existed with destruction of the periosteum for the whole length. On inspecting the bone, a hole, about the size of a lead pencil, was found in the internal condyle leading down to the hollow and from it pus exuded.

I then extended my incision and laid bare the femur for about nine inches, or the whole length of the cavity. This was not easy to accomplish on account of the proximity to the femoral artery. Then with a chisel, I grooved the femur for about eight inches and thoroughly scraped out the decomposed medullary substance from the shaft and hollowed the internal condyle. I then made a large opening on opposite side of leg—washed thoroughly and packed with iodoform gauze.

The operation was long and tedious, and this, coupled with his extremely bad condition when I began, caused the patient to be taken from the table pulseless.

Stimulants hypodermically and hot applicants, however, brought about a reaction.

His temperature that night rose to 104 2-5 F., but gradually fell to 99 F. by the next morning, which was the lowest point for weeks previous.

The dressings were removed in 48 hours and the parts irrigated with sterile water and peroxide of hydrogen. The wound looked splendidly. The general condition of the patient improved rapidly. Everything seemed to go nicely for about four weeks, when the fever suddenly ran high and the knee became red, tender and swollen.

On August 10th, with the assistance of the above named gentlemen, I reopened the old cicatrix and fistula and found there had been much improvement in the upper part of the femur; but the disease of the internal condyle had gone closer to the knee joint. In fact it was impossible to absolutely determine whether or not the disease had eaten into the joint. It looked as if an amputation of the leg in the middle third of the thigh was almost inevitable. I was determined, however, not to sacrifice the limb so long as there was a chance to save it, and as his general condition was now so much better than at the time of his first operation, I limited my procedure to very thoroughly curetting all the diseased bone, then packed and dressed as before.

Peroxide of hydrogen—Boracic Acid—Iodoform and Balsam of Peru were in turns used to stimulate granulations.

On September 19th, Dr. Luter again administered a little chloroform and I scraped away some false granulations of the soft parts and enlarged the external openings for better drainage. Soon afterwards I put the boy on crutches and into the open air and sun. Since that time he has made gradual but substantial improvement.

The drainage now is but little—the cavity small—soreness gone, and I believe that the patient is going to get entirely well and have a useful leg.

Drs. Edmondson and Luter dressed the case while I was absent on my vacation.

I report this case not thinking that osteomyelitis is a rare disease, but to show the necessity of a correct diagnosis and an early operation.

This case ought to have been recognized during the first few days of its existence, and certainly not later than the time when the periosteum gave way and allowed the pus to extravasate between the bone and muscles.

A thorough operation at that time would have insured an easy and rapid cure.

My second case is one of Mrs. J., aged 21.

Girlhood health had been reasonably fair. Been married about fifteen months. During April of this year, when about four months pregnant, she was suddenly taken with severe pains in right side towards the back. I am informed that the physicians who saw her pronounced the case one of acute appendicitis and advised an operation, but it was refused.

The patient suffered very much, and was in bed about fifteen days.

I was called to see her for the first time on September 11th. The nine months of pregnancy was about up, and I found her with crampy pains over the whole abdomen, such as usually precede true labor. These continued for forty-eight hours, when expulsive pains began, and she was delivered of a dead fœtus at full term.

The fœtus must have been dead for several days, as the epidermis easily slipped off on handling. The placenta was quite adherent, and so I inserted my hand into the uterus and cleaned it out. Two hours afterwards I used my thermometer and found that she had fever. For eight or nine days it ranged from 100 to 102 F. I was satisfied that this elevation in temperature was not from the uterus, as the lochia was quite normal. During all these days there were uneasy, gripy pains over the lower part of the abdomen. These pains were increased in severity, and on the ninth day after confinement the abdomen was everywhere quite sensitive and much enlarged on the right side over the cæcum. The fever had reached 103 F.

On the next day, September 22nd, the tumefaction had grown very much larger and more sensitive; with a morning temperature of 103 2-5 F.

Dr. Hicks saw the case in consultation, and both of us believed it one of ruptured appendix with a large quantity of pus walled off by adhesions from the general peritoneal cavity. The tension seemed so great that I feared the sac would rupture at any hour—I did not dare move the patient from the house. She was so weak and exhausted that it looked almost like a hopeless case.

I made the necessary arrangements and operated at 4:30 on the afternoon of September 22nd. My incision was an oblique one, and well over on the outer side of the tumefaction.

I opened down to the peritoneum and then inserted an operating needle into the tumor and drew off black offensive blood. I then made a free incision and emptied about one quart of liquid and clotted blood with a great deal of fibrin or membraneous tissue, I could not be certain which.

The entire contents of the sac was black and extremely offensive, showing that it had been thus accumulated for a long time.

The cavity was completely walled off by the muscles and skin externally—the liver above—omentum and intestines internally and the broad ligament formed the floor below. The fimbriated end of the fallopian tube floated free in the cavity, and showed through the incision. The central portion of the tube was surrounded by a mass of adhesions.

The cavity was thoroughly washed with sterile water and packed with iodoform gauze.

The fever dropped to 99 F.—the pains subsided and the patient began at once to improve. For two or three days the cavity walls felt hard and clean, but soon the fibrinous or membraneous clots began to loosen and come away. The offensive discharge increased, and the fever ran some higher. Whenever I caused the bowels to move with salines and enæmas the fever would afterwards rise—due, I believe, to the increased absorbing power of the system.

On the sixth or seventh day after the operation, and just following a brisk purge, she had a hard chill. The temperature ran up to 106 1-5 F., but remained high for only a few hours.

The cavity became free of offensive clots about two weeks after the operation, and since then has contracted and healed, until it is now nearly closed.

My patient is free of pains—without fever and able to sit up.

I am under obligations to Drs. Hicks, Luter and Earp, who rendered me valuable assistance.

The query now is, from whence came the hemorrhage and at what time did it occur?

I think it came through the fallopian tube, but whether it was caused by its rupture or as a free hemorrhage from the uterus, is more than I can tell.

There was no fœtus found to positively prove the presence of an ectopic pregnancy—yet it is my belief that double pregnancy took place at the same time—one in the uterus and the other in the fallopian tube. From some cause death must have occurred early to the tubal fæcundation, but I believe the rupturing and emptying of the tube took place at the time of her illness in April, when the severe pains existed. Under these conditions the blood would be sterile, and that acounts for why this large accumulation of blood could remain saculated from April to September without causing more inconvenience and disturbance than it did. The pregnant condition did not allow the tumor to show, but pushed it backward and upward until after the confinement, when room was made for it in the abdomen. It must have become infected, however, from its long contact with the intestines which formed the greater part of the internal wall of the cavity.

This case, I think, proves the value of conservative surgery, for if I had attempted to remove the sac, the general peritoneal cavity would have become infected and the life of my patient been sacrificed.

